# Evaluation of deep convolutional neural networks for automatic classification of common maternal fetal ultrasound planes

**DOI:** 10.1038/s41598-020-67076-5

**Published:** 2020-06-23

**Authors:** Xavier P. Burgos-Artizzu, David Coronado-Gutiérrez, Brenda Valenzuela-Alcaraz, Elisenda Bonet-Carne, Elisenda Eixarch, Fatima Crispi, Eduard Gratacós

**Affiliations:** 1 BCNatal - Barcelona Center for Maternal-Fetal and Neonatal Medicine (Hospital Clínic and Hospital Sant Joan de Deu, University of Barcelona), Barcelona, Spain; 2 Transmural Biotech S.L., Barcelona, Spain; 3 Institut D’Investigacions Biomediques August Pi i Sunyer, IDIBAPS, Barcelona, Spain; 4 Center for Biomedical Research on Rare Diseases (CIBER-ER), Madrid, Spain

**Keywords:** Ultrasonography, Translational research, Computer science

## Abstract

The goal of this study was to evaluate the maturity of current Deep Learning classification techniques for their application in a real maternal-fetal clinical environment. A large dataset of routinely acquired maternal-fetal screening ultrasound images (which will be made publicly available) was collected from two different hospitals by several operators and ultrasound machines. All images were manually labeled by an expert maternal fetal clinician. Images were divided into 6 classes: four of the most widely used fetal anatomical planes (Abdomen, Brain, Femur and Thorax), the mother’s cervix (widely used for prematurity screening) and a general category to include any other less common image plane. Fetal brain images were further categorized into the 3 most common fetal brain planes (Trans-thalamic, Trans-cerebellum, Trans-ventricular) to judge fine grain categorization performance. The final dataset is comprised of over 12,400 images from 1,792 patients, making it the largest ultrasound dataset to date. We then evaluated a wide variety of state-of-the-art deep Convolutional Neural Networks on this dataset and analyzed results in depth, comparing the computational models to research technicians, which are the ones currently performing the task daily. Results indicate for the first time that computational models have similar performance compared to humans when classifying common planes in human fetal examination. However, the dataset leaves the door open on future research to further improve results, especially on fine-grained plane categorization.

## Introduction

Ultrasound (US) examination is an essential tool to monitor fetus and mother along pregnancy, providing an economic and non-invasive way to observe the development of all fetal organs and maternal structures. Several measures obtained from maternal-fetal scans are commonly used to monitor fetal growth^1^. The most commonly used biomarkers in clinical practice for the screening of fetal abnormalities are fetal biometries, estimates of fetal weight, and/or Doppler blood flow^2^. For example, nuchal translucency measurement is the basis for the first trimester screening of fetal aneuploidies^3^, estimated fetal weight is used to detect abnormal growth^4^, fetal lungs can be used to predict neonatal respiratory morbidity^5^ and uterine cervix can be used to determine the risk of a preterm delivery^6,7^.

The acquisition of fetal and maternal ultrasound images in most fetal medicine centers is done following international guidelines promoted by scientific committees^8,9^. This means that images are obtained following the same protocols in a repeatable way. Indeed, images need to be acquired in a specific plane to be useful for diagnosis, to decrease the inter- and intra-observer variability and to allow the measurement of specific structures. Typically, more than 20 images are acquired for each ultrasound examination within mid-trimester screening ultrasound^8^. Occasionally, three dimensional (3D) images and videos can also be acquired to complete the clinical examination.

Both in a clinical setting and in research projects, a fetal specialist reviews the sonographer’s examinations, selecting images containing the structures of interest. Usually, trained research technicians, followed by a validation from a senior maternal-fetal expert, manually perform this task. However, since each screening ultrasound examination contains more than 20 images (55 in average in our hospitals), this process is slow, cumbersome and sensitive to mistakes. Therefore, an automatic system performing this task would increase cost-effectiveness and may reduce errors.

Artificial Intelligence has undergone impressive growth during the last decade, in particular with the emergence of Deep Learning (DL)^10^ and its remarkable progress in image recognition tasks via Convolutional Neural Networks (CNNs). In the last few years, CNNs have shown its usefulness in a wide set of medical applications, such as dermatology^11^, radiology^12^ and/or to classify or segment organs and lesions in images from computer tomographies and MRI^13^. These methods are known to excel *“at automatically recognizing complex patterns in image data and provide a quantitative, rather than qualitative assessment”*^12^.

However, in comparison, the use of CNNs in US remains limited to date^13^. And even more so prior work at using CNNs to select or classify US planes. The majority of this work detects US planes of interest from 2D^14,15^ or 3D^16,17^ video data. For example^14^, was the first to use CNNs for real-time automated detection of 13 fetal standard scan planes, the method uses weak supervision based on image level labels. The study used very rich data, with carefully acquired videos longer than the usual ones acquired in clinical practice. Each US video was approximately 30 minutes long, which provided surrounding and additional information to the CNNs. In^15^, authors used conditional random field models to detect the fetal heart in each frame of the 2D video. Video data information was used to take into account the temporal relationship between the frames^16^. proposed an hybrid method, which uses Random Forests to localize the whole fetus in the sagittal plane and, then CNNs to localize the fetal head, fetal body and non-fetal regions (in axial plane images). To obtain the best fetal head and abdomen planes the method uses clinical knowledge of the position of the fetal biometry planes within the head and body. Finally^17^, proposed an iterative approach with multiple passes of CNN to detect standard planes in 3D fetal brain US.

Although ultrasound studies are a real-time evaluation with recognition of the planes and structures from real-time moving images, currently, relying in video or 3D data limits its use for retrospective studies, since videos and/or 3D US are not always performed or saved. The majority of data stored in fetal clinics are 2d still images, for several reasons such as including standard planes for systematic measurement of fetal biometry, recording a representative view of a real-time evaluation of fetal anatomy to demonstrate that this has been assessed as normal, and the existing cost of storage of large volumes of data in PACS systems. In our hospitals, currently less than 1% of the patients have videos associated. With increasing improvements in technology there will be an increased storage of videoclips in fetal imaging, but we are still far.

For this reason, we believe that recognition of 2d fetal images without relying on video is still of adamant importance for current clinical studies. Moreover, improving recognition and detection on images is the first pillar to improve the technology in general. These improvements will be the foundation for future tools that can assist the examination in real-time. In this study we release publicly a large dataset of images and a large evaluation of CNNs to encourage research on this field and move us closer to the goal of creating better A.I. tools for maternal-fetal medicine.

Recently, Cheng and Malhi^18^ demonstrated that transfer learning with CNNs can be used to classify abdominal 2D ultrasound images into 11 categories. They evaluated two CNNs (CaeNet and VGGNet) and compared their performance against that of a radiologist. In our work we follow a similar approach but applied to maternal-fetal US, which has its own particularities and is quite different from standard abdominal US. Furthermore, we perform a much larger evaluation and improve significantly overall recognition performance.

In this context, the goal of this study was two-fold: (1) evaluate the maturity of state-of-the-art CNNs to automatically classify 2D maternal fetal US and (2) the release of a large open-source dataset to promote further research on the matter. With this purpose in mind, we first collected a large maternal-fetal 2D US dataset. All images were manually labeled by two research technicians and a senior maternal-fetal clinician (B.V-A.). Finally, an exhaustive evaluation of state-of-the-art DL methods was performed using a benchmark protocol mimicking a real scenario, to judge how mature the technology is for its use in everyday clinical practice.

**Figure 1:**
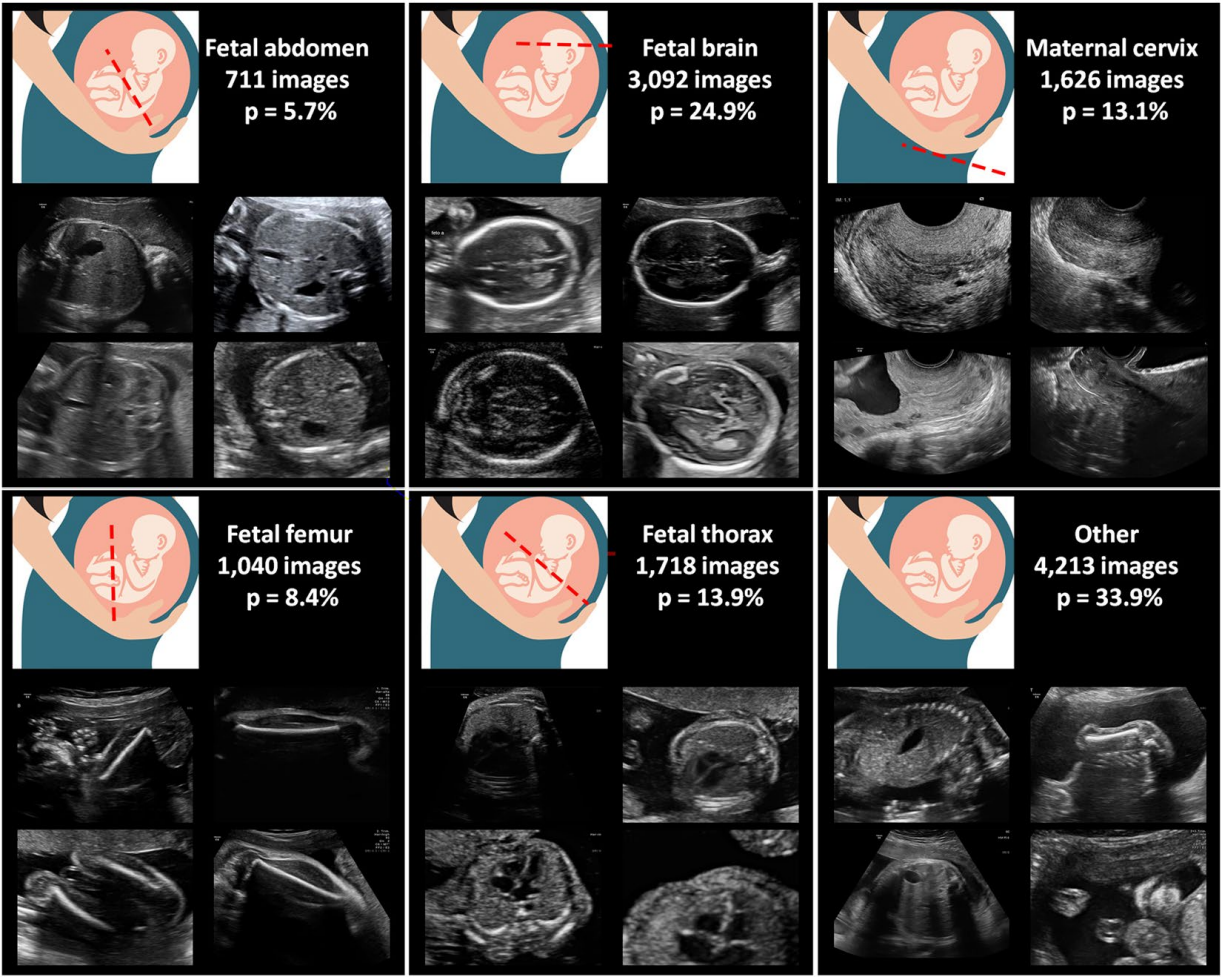
Maternal-fetal US categories from our dataset: image examples, total number of images and frequency of occurrence (p). Fetus drawing was downloaded from openclipart.org, a creative commons repository of images for public domain use.

**Figure 2:**
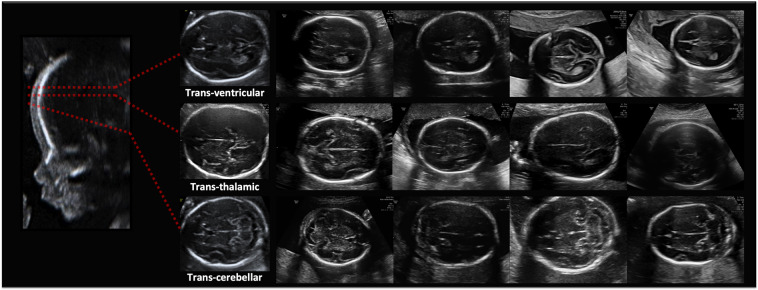
Fine-grained brain labels: image examples.

The contributions of this study are three-fold:

1. The collection of a large maternal-fetal ultrasound image dataset comprised of more than twelve thousand images from 1,792 patients in a real clinical setting. All images were labeled with 5 + 1 of the most widely used maternal-fetal anatomical planes by a senior maternal-fetal clinician, and further augmented with fetal brain planes, see Figs. 1 and 2 and Table 1. The dataset is the largest US dataset to date to our knowledge and represents a real clinical scenario with unbalanced data. The dataset will be made publicly available upon publication of this paper, to promote research on automatic maternal-fetal US recognition methods.

**Table 1:** Maternal-fetal US dataset statistics: anatomical planes labeled, number of patients, number of images, US machines and Operators.

Anatomical plane	Clinical use			N. patients	N. images
Fetal abdomen	Morphology, fetal weight			595	711
Fetal brain				1,082	3,092
Trans-thalamic	Neuro-development,			909	1,638
Trans-cerebellum	fetal weight			575	714
Trans-ventricular				446	597
Fetal femur	Fetal weight			754	1,040
Fetal thorax	Heart and lung development			755	1,718
Maternal cervix	Prematurity			917	1,626
Other	Several			734	4,213
TOTAL				1,792	12,400
**US machine**	**N. patients**	**N. images**	**Operator**	**N. patients**	**N. images**
Voluson E6	807	5,862	Op. 1	407	2,792
Voluson S10	91	1,082	Op. 2	344	2,435
Aloka	270	3,560	Op. 3	270	3,560
Others	631	1,896	Others	803	3,613
2. A comprehensive evaluation of different state-of-the-art CNNs on the newly collected dataset, see Table 2. The benchmark protocol was designed to mimic a real scenario: images were separated by patient and study date, using the first half of the patients to train and the next half for testing. Further scenarios were also evaluated for completeness.

**Table 2:** Results of the wide variety of classification CNN tested for maternal-fetal common planes recognition.

Net	Params	Layers	Speed (Hz)	top1-err(%)	top3-err(%)	class-acc(%)
VGG-M^22^	99 M	25	110	12.9	0.66	84.8 +− 9.3
VGG-16^22^	134 M	54	40	7.9	0.55	92.1 +− 6.1
MobileNet^25^	2 M	157	20	10.7	0.82	87.5 +− 9.6
Inception-v3^24^	22 M	318	12	6.5	0.34	93.5 +− 5.0
ResNet-18^23^	11 M	73	66	7.6	0.47	92.5 +− 5.9
ResNet-34^23^	21 M	129	38	7.6	0.76	92.5 +− 5.8
ResNet-50^23^	24 M	177	25	6.8	0.32	93.1 +− 5.4
ResNet-101^23^	43 M	347	10	6.7	0.23	93.4 +− 5.2
ResNet-152^23^	58 M	517	5	6.5	0.21	92.8 +− 5.5
ResNeXt-50^26^	23 M	179	25	7.3	0.34	92.7 +− 5.9
ResNeXt-101^26^	42 M	348	13	6.5	0.55	94.0 +− 4.8
SENet^27^	113 M	773	2	7.6	0.27	92.9 +− 5.9
SE-ResNet-50^27^	26 M	256	14	7.6	0.76	93.3 +− 6.1
SE-ResNet-101^27^	47 M	511	6	7.0	0.36	93.3 +− 6.1
SE-ResNet-152^27^	47 M	511	3	7.5	0.32	92.7 +− 6.0
SE-ResNeXt-50^27^	25 M	256	15	7.1	0.21	92.7 +− 5.7
SE-ResNeXt-101^27^	47 M	511	6	7.1	0.27	92.7 +− 5.8
DenseNet-121^28^	8 M	428	11	7.1	0.32	92.9 +− 5.8
DenseNet-169^28^	14 M	596	7	6.2	0.27	93.6 +− 5.1
Baseline1 (PCA + Boosting)	—	—	41	39.6	10.4	54.7 +− 37.6
Baseline2 (Hog+Boosting)	—	—	28	25.5	5.2	68.6 +− 28.8

**Figure 3:**
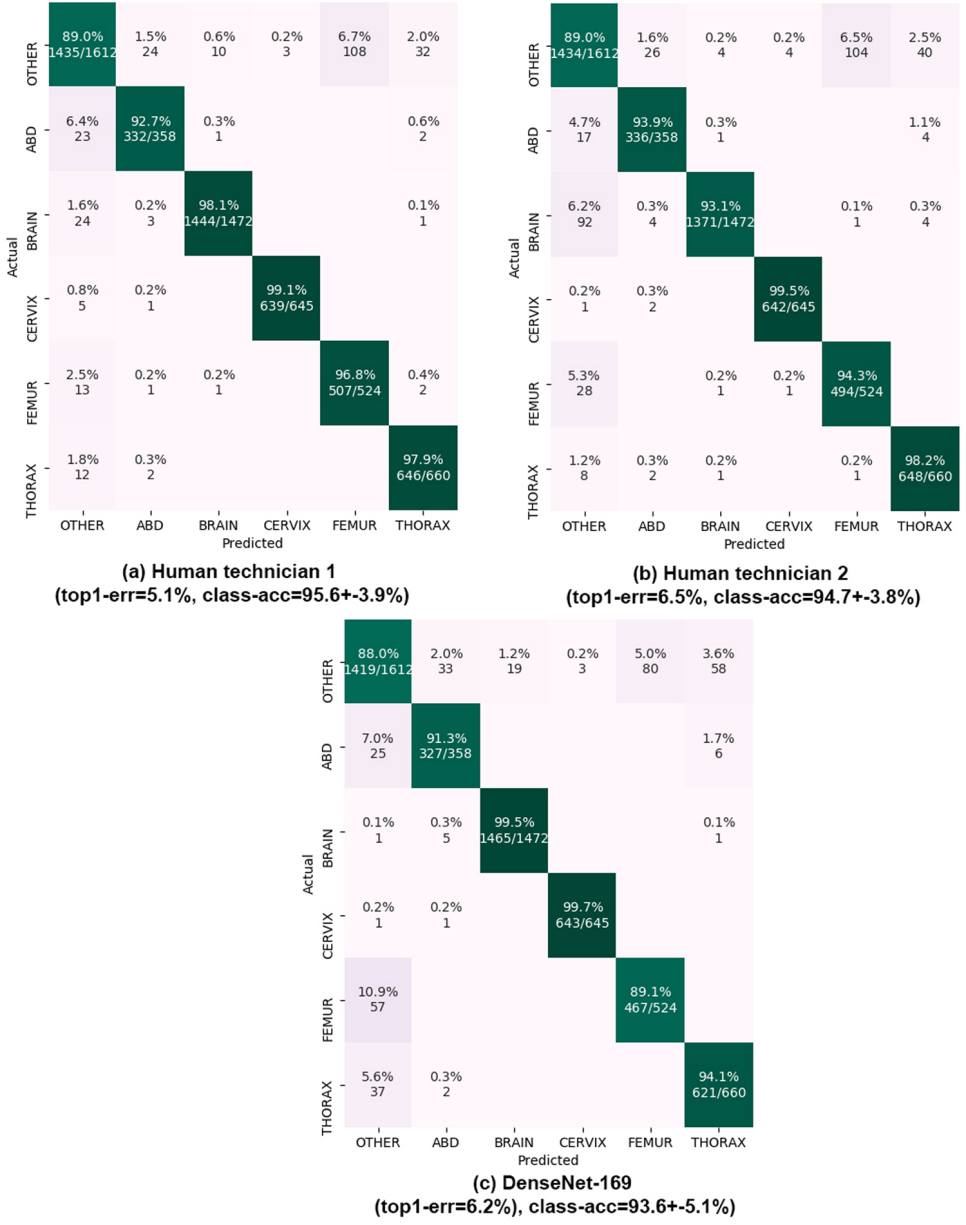
Results on common planes classification. Confusion matrices on the 896 test patients (5,271 images) are shown. Matrix rows show the true class, labeled by our expert maternal-fetal clinician. Top-1 error and mean +− std of the diagonal (class-acc) are shown. Matrix columns are the prediction from (**a**,**b**) our two research technicians and (**c**) DenseNet-169 model.

**Figure 4:**
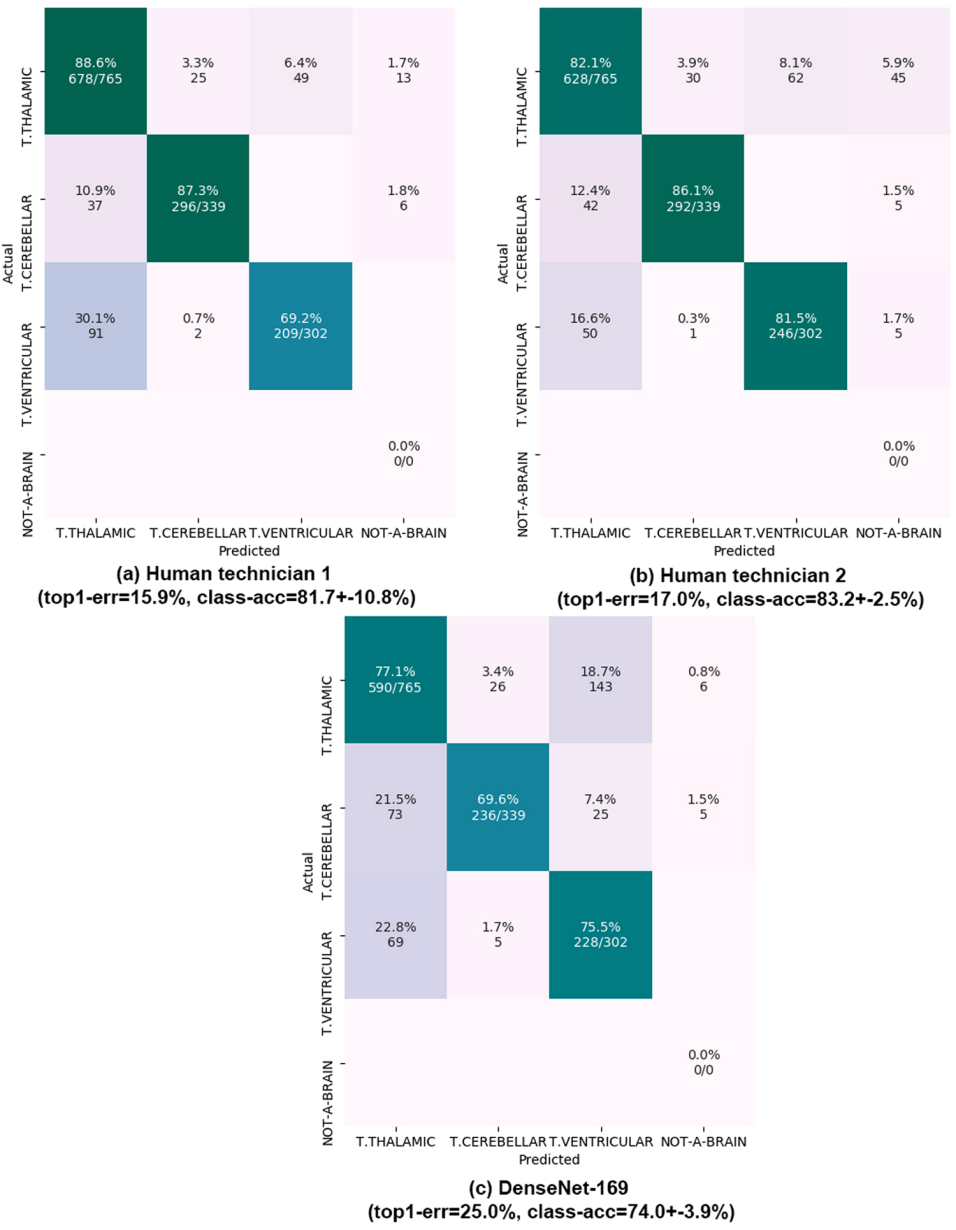
Results on fine-grained brain categorization. Confusion matrices on the 536 test patients (1,406 images). Matrix rows show the true class, labeled by our expert maternal-fetal clinician. Top-1 error and mean +− std of the diagonal (class-acc) are shown. Matrix columns are the prediction from (**a**,**b**) our two research technicians and (**c**) DenseNet-169 model. All numbers are shown as percentages. NOT-A-BRAIN: mistaken by something other than a Brain.

DenseNet-169 is the best performing model in terms of top-1 error. Inception and ResNetXt-101 are the best performing models taking into account trade-off between speed and performance.
3. The direct comparison between state-of-the-art DL techniques and the classification performed by research technicians who perform the task daily in our hospitals, see Figs. 3 and 4. This comparison allows to judge the maturity of the technology and pinpoint areas that need improving, promoting future research. Results suggest for the first time that computational models can be used to classify common planes in human fetal examination.

## Materials and Methods

### Study design

The dataset was collected at BCNatal, a center with two sites (Hospital Clinic and Hospital Sant Joan de Deu, Barcelona, Spain), with large dedicated maternal-fetal departments handling thousands of deliveries per year. Images were acquired during standard clinical practice between October 2018 and April 2019. All pregnant women attending for routinary pregnancy screening during second and third trimester were included in the study. Multiple pregnancy, congenital malformations or aneuploidies were excluded. Gestational age was computed from crown-rump length measurements on first-trimester US^19^ and ranged from 18 to 40 weeks. All the methods hereby explained were performed in accordance with the relevant guidelines and regulations and approved, together with the study protocol by the coordinator’s Institutional Review Board (Comite de etica de investigacion clinica, ID HCB 2018/0031). All patients provided written informed consent to use US images for research purposes.

### Image acquisition and labeling

Images were acquired from a total of six different US machines by several different operators with similar experience. US machines were three Voluson E6 (GE Medical Systems, Zipf, Austria), one Voluson S8, one Voluson S10 (GE Medical Systems, Zipf, Austria) and one Aloka (Aloka CO., LTD.). Images were taken using a curved transducer with a frequency range from 3 to 7.5 MHz (abdominal US) or a 2 to 10-MHz vaginal probe (used for cervical US screening in second trimester patients). Operators were instructed to avoid using any type of post-processing or artifacts such as smoothing, noise, pointers or calipers when possible. The remaining image settings parameters such as gain, frequency and gain compensation were left to their discretion. All images were stored in the original Digital Imaging and Communication in Medicine (DICOM) format.

In average each US study was comprised of 55 images. A Graphical User Interface (GUI) was developed in python (Python Software Foundation, USA) to allow a senior maternal-fetal specialist (B.V.-A.) to manually classify the images. The clinician selected images belonging to the five anatomical planes most widely used for maternal-fetal during fetal routine screening, see Table 1. Only images complying with the minimal quality requirements were selected by the clinician, excluding those with inappropriate anatomical plane (cropped or badly taken) and those with calipers. The dataset composition is clearly unbalanced (some classes are much more frequent than others) as is usually the case in real clinical scenarios.

Once all images labeled, in order to benchmark the performance of computational models on more fine-grained categorization, we further asked our clinical expert to extend labels of brain images with the specific brain plane, divided into the three most common planes: Trans-ventricular, Trans-thalamic, and Trans-cerebellar. These three planes together account for 95% of all brain images, see Table 1.

Finally, two research technicians were asked to independently classify images from the second half of patients (test images, see below). These two research technicians were not clinical experts, but received extensive training in US classification. The performance of both technicians is used as baseline performance on the dataset, to judge the maturity of the computational methods for the automatization of the task.

### Final dataset & data public release

To be able to measure false discovery rates of the methods benchmarked, an additional category called “Other” was created. This category contains a random subset of images not previously selected by the expert clinician, but avoiding images from the five main categories that were discarded for quality reasons (anatomical plane cropped or calipers). A total of 4,213 such other images were added; the final dataset being composed of 12,400 images.

To avoid ethical issues, all patient data from original DICOM images were fully removed by removing image header and images were stored in PNG (Portable Network Graphic) format (without compression to avoid losing quality). Since US images do not convey color information, images were stored as grayscale bitmaps.

The released dataset will contain all the information used in this study, namely:

1. All 12,400 images without header, in png format.
2. Fetal anatomical labels for each image (5 + 1 categories).
3. Fine-grained brain anatomical labels (3 categories) for each brain image.
4. Anonymized patient IDs for each image, where IDs are a consecutive 4 digit number ordering patients in ascending chronological order according to their first visit.
5. Training and Testing patients used in this paper.
6. US machine and Operator IDs for each image.
7. The two manual re-classification from both research technicians on the test images.

The dataset will be hosted in a dedicated website, where we will also maintain a list of published papers that use the dataset, reporting performance improvements.

### Methods used

The goal of the study was to benchmark current classification techniques on the new dataset. In order to do so, and to mimick a real scenario, we ordered patients chronologically by the date of their first visit, and used all images from the first half of patients to train the classifiers and images from the second half of patients to evaluate the methods and report their performance. This resulted in a training set containing 7,129 images and a testing set containing 5,271 images (both with 896 patients).

#### Simple baselines

To judge the difficulty of the task, we first evaluated two simple non-DL classifiers. Both use as learning algorithm a multi-class Boosting algorithm^20^. To process the images, the first one simply applies Principal Component Analysis to the image pixels, while the second uses classical Histogram of Oriented Gradients (HOG) features^21^.

#### CNN classifiers

Then, we benchmarked a large set of the most widely used state-of-the-art classification CNNs, including many different architectures and a wide variety of depths, number of total parameters and processing speeds^22–28^.

In all cases, the networks’ original architecture was maintained, and the nets were trained following the original author’s guidelines. All networks were first pre-trained on ImageNet Large Scale Visual Recognition Challenge^29^, and then fully retrained (allowing changes to the entire network) using our training data. After training, the nets were frozen and applied to the test images, outputting full probability scores for each class.

**Implementation details** All nets were trained following the original author’s guidelines using softmax cross-entropy loss and adam optimizer. 10% of the training set was used as validation set. Nets were allowed to train for a maximum of 15 epochs, early stopping if loss on validation set was not improved for 5 consecutive epochs. Learning rates were adjusted differently according to the network following its original author’s guidelines. Batch size was 32 and weight decay was 0.9. To improve learning, data augmentation was used during training. At each batch, images were randomly flipped, cropped between 0–20%, translated from 0–10 pixels and rotated between [−15; 15] degrees.

ResNets and DenseNets were implemented in TensorFlow from official models^30^ and pretrained on ImageNet Large Scale Visual Recognition Challenge^29^ for 90 epochs with a 5 epoch warm-up. The rest were implemented in MatConvNet^31^ and pre-trained Imagenet models were directly downloaded from^32^. Input image size was [224 × 224] for all nets except for Inception which uses a [299 × 299] image size. Since images were grayscale, the first convolutional layer of all nets was adapted to work on 1 channel after training on Imagenet, using the average of each one of the first 3 convolutional channel filters. Lastly, Fully-Connected layer of each network was adapted to output 6 classes instead of ImageNet’s 1,000. Batch-normalization^33^ was used for all nets after convolution layers. No further weight regularization was enforced. Input US images were converted from integer in range [0, 255] to single float precision in [−1, 1] range using the norm of all training images.

### Statistical analysis

#### Main results

Each one of the methods was applied to the test images, storing full probability scores. Then, top-1 error and top-3 errors with respect to test labels produced by our clinician were computed. Top-1 error (1-accuracy) indicates the percentage of miss-classified images and was considered the main metric. Top-3 error was used to judge how often the true class was predicted as one of the 3 most likely classes (out of the 6 total classes), to judge the net’s margin on miss-classifications. Then, full confusion matrices were also computed and the mean and standard deviation of the diagonal of the confusion matrix was reported to analyze average accuracy on each class. This same process was repeated also to compare labels from the two research technicians with those of the clinician (except top-3 error could not be computed in this case). Main results are shown in Table 2, and full confusion matrices of both the research technicians and the best CNN are shown in Fig. 3.

#### Fine-grained brain labels

We used the best performing model CNN according to Table 2 results (Densenet-169) to further test performance on fine-grained brain classification. We used the original training and testing patients, keeping only those that had at least one image of the three brain sub-planes. The result was a train set composed of 592 patients and 1,543 images, and a test set having 536 patients and 1,406 images. Then, the training set was augmented with 500 random images from other planes. The net was trained and tested on the new data, using the same test images to report research technicians’ performance. Results are shown in Fig. 4.

#### Ablation study

For completeness, using one of the nets (Inception) showing best trade-off between speed and performance according to Table 2 results, we performed an ablation study to analyze the effect that some technical details have on performance. The following was evaluated:

1. Different CNN training: We tested performance vs some training parameters, such as not pre-training models on ImageNet, performing only fine-tuning of the last layer, removing data augmentation and using a data balancing approach^34^. Results are shown in Table 3.

**Table 3:** Ablation study results using Inception.

Experiment	top1-err(%)	top3-err(%)	class-acc(%)
No Imagenet pre-training	14.1	0.89	84.7 +− 11.6
Last layer training only	10.7	0.57	87.6 +− 9.7
No data augmentation	8.1	0.66	92.2 +− 6.6
Data reweighting^34^	7.4	0.36	92.4 +− 6.0
Baseline	6.5	0.34	93.5 +− 5.0
2. Performance vs number of train patients/images: We tested performance vs number of training patients/images. We created 8 additional training sets by removing from the original training set a hundred patients at a time, and each was used to train the CNN. Testing was performed on the same, unchanged test dataset containing 896 patients. Results are shown in Fig. 5.

**Figure 5:**
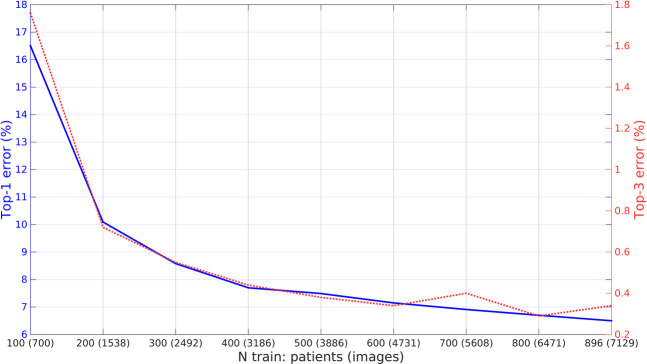
Performance vs number of training patients/images. Performance of the Inception CNN on the test set as a function of the number of training patients used.
3. Transferability: We created several different training and test sets, partitioned by which US machine and operator had collected each image, using only US machines and Operators that have at least 2,000 total images. The CNN (Inception) was trained and tested on each set and compared with normal model trained on images from all US machines and operators. Results are shown in Table 4.

**Table 4:** Model transferability between US machines and operators.

Training (N images)	Testing (N images)			
	Operator 1 (874)	Operator 2 (946)	Operator 3 (1,460)	ALL (5,271)
Operator 1 (1,918)	9.7	13.0	33.0	18.9
Operator 2 (1,489)	17.5	10.6	32.7	23.0
Operator 3 (2,100)	24.8	24.4	4.3	19.6
ALL (7,129)	7.2	7.5	3.7	6.5

Images were divided into categories according to US machine and operator, keeping those having at least a total of 2, 000 images. Then, each category was used to train an Inception CNN (table rows) and tested on sets built using images from all categories (columns). Numbers are top-1error in the test set (%). Operators 1 and 2 always used a Voluson E6 US machine, while Operator 3 used exclusively an Aloka US machine.

## Results

### Maternal-fetal US dataset

Table 1 summarizes the characteristics of the final dataset. A total of 1,792 patients were recruited. Some of them had a longitudinal follow-up, having a total of 2,087 distinct US studies. Images were classified into the five anatomical planes most widely used for maternal-fetal during fetal routinary screening, plus an extra category called “Other” containing other planes. Furthermore, brain images were categorized into their sub-plane using three categories. Images were collected from several ultrasound machines and by different operators. The dataset is comprised by a total of 12,400 images. Since images were collected prospectively from a real clinical scenario by different US machines and operators, classes show high variability and have different probabilities of appearance, see Table 1. Figures 1 and 2 show visual examples of the type of US images present in the dataset for each category.

### Classification results

Table 2 shows the main results of each method on the test set, comprised of 5,271 images from the second half (896) of patients. Best performing net is DenseNet-169 with a 6.2% top- 1 error, 0.27% top-3 error and 93.6% average class accuracy. Taking into account the trade-off between performance and speed/size, both Inception and ResNetXt-101 achieve similar performance (6.5% top-1 error) while doubling DenseNet-169’s speed. In general, variation in performance is low, with the difference between lowest and highest top-1 errors being 6.7%.

More modern architectures do not always perform better than older ones: while DenseNets work well, Squeeze-Excitation variants of classical ResNet networks sometimes perform worse than the classical architectures. As for depth, deeper nets usually perform better in general. The non-DL baselines are clearly much weaker, reaching top-1 errors above 25%.

#### Ablation study

Using one of the nets showing best trade-off between speed and performance in previous section (Inception), we performed an ablation study to analyze the effect that some technical details have on performance. Table 3 and Fig. 5 show the results:

1. No pre-training: If the network is directly trained from scratch on our dataset, top-1 error increases 8% when compared with using a pre-trained Imagenet model. This shows the importance of pre-training models on a very large dataset and that convolutional filters learned on natural images are indeed transferable to US.
2. Last layer training only: If the pre-trained network is only fine-tuned (only last convolutional branch and final fully connected classification layer are allowed to change) top-1 error increases 4% when compared with a full re-training of the net. This shows that re-training on our dataset is more important on deeper layers, but that allowing the entire net to change is beneficial.
3. No data augmentation: Similar to what observed in natural images, data augmentation during training is beneficial: removing it makes top-1 error increase a 1.6%.
4. Data balancing: Using a class re-weighting approach^34^ during training to help the model with class unbalance did not improve performance, in fact top-1error increased slightly by 1%.
5. Number of training images/patients: Fig. 5 shows the performance of Inception model on the test set as a function of the number of patients used for training. Errors drop significantly as soon as a few hundred patients are seen during training, but maximum performance is reached only when all images are used.

#### Computational model transferability

A key factor for the applicability of automatic computational methods in medicine is the transferability of results between centers, different equipment and operators^35^. We tested how well models built using images exclusively from one machine/operator can translate to the rest. Results are shown in Table 4, where rows correspond to the type of images used for training and columns to the type of test images. This experiment had two relevant results:

1. It is always preferable to train the model on images from all sources (all US machines and operators). Even when evaluating on images from a specific US machine/operator, a model trained from all sources yields better performance than a model trained specifically for that US machine/operator.
2. Both US machines and operators have an important impact in performance. However, of the two, US machines have by far the most important impact. Clearly, the computational model can quickly overfit to the type of US images seen, and results when trained only on a machine and tested on a different can show almost a two-fold increase in error rates.

#### Comparison vs human

We directly compare the performance of the best computational model (DenseNet-169) against that of our research technicians, using images from all 896 test patients. Full confusion matrices for the research technicians and best computational model are shown in Fig. 3. Both achieve similar performance, with top-1 errors of 5.1% and 6.5% for the technicians and 6.2% for the CNN. The CNN reaches close to perfect performance on 2 of the 6 classes (Brain and Cervix), but performs slightly worse than both technicians on the other 4 classes (Other, Abdomen, Femur and Thorax). Femur seems to be especially hard for the CNN; we believe this is due to the fact that Other category contains other bones (Humerus, Radius, Tibia, etc.) which can look very similar. As for processing time, the computational model processes images at 7 Hz (0.14 seconds per image), while research technicians require an average of 3.5 seconds to classify each image using our GUI.

#### Fine-grained brain categorization

Finally, we also evaluate performance of the best computational model (DenseNet-169) on fine grained brain categorization and compare its performance against that of our research technicians, using all brain images from our test patients (536 patients, 1406 images). Results are shown in Fig. 4. Errors are much higher than before, showcasing the difficulty of the task. Technicians reach top-1 errors of 15.9% and 17.0% respectively, while the CNN has a 25.0% error. In this case, the computational model has worse performance in all three classes compared to research technicians.

## Discussion

In this study we have extensively evaluated current state-of-the-art CNNs for the task of maternal-fetal US classification. For the first time, a large dataset of maternal-fetal US was collected and carefully labeled by an expert clinician. A wide variety of CNN were benchmarked using a setting mimicking a real clinical scenario and their performance directly compared on the same data against research technicians who performs this task on a daily basis.

The dataset is a good representation of a real clinical setting since images were collected prospectively by different operators using several US machines. Classes show high intra-class variability and dataset composition is clearly unbalanced, see Fig. 1 and Table 1. As far as we know, the dataset is the largest US dataset to date publicly available to promote research on automatic US recognition methods.

Results on general classification show that current state-of-the-art computational models developed for general visual recognition from standard photographs can also work for maternal-fetal US recognition. The best performing computational model was able to classify US images almost on par with research technicians fully trained to perform the task, meaning that the for the first time this technology is reported to be mature enough as to be applied inside a real clinical circuit for classifying common planes in human fetal ultrasound examination. Furthermore, the computational model classifies images 25× faster and can work 24 h/7, meaning its use could result in an increased cost-effectiveness compared to using technicians. However, considering the wide variety of configurations benchmarked and the narrow variation in performance, we also believe that current technology is reaching a saturation point. Researching novel methods tailored for its use on US images might still be worthwhile to expand these promising results to other tasks.

Results on brain fine-grained categorization show another scenario altogether: computational models are still far from human technicians on this (much harder) task, where small details make all the difference. Clearly, further research is needed in this area. Another concern is the transferability of computational models. As shown by results, a computational model learned using exclusively images from one operator or US machine cannot be directly transferred to images from other operators or US machines. This is a key issue of computational models, which cannot be ignored in the medical field^35,36^. The small size of public image datasets has been so far a limiting factor. We hope that the release of the dataset will help researchers worldwide and promote further research in this area.

This study has several strengths. We collected the largest US dataset to date, as far as we know. The dataset represents well a real clinical scenario and we hope its public release will encourage future work on this data. We evaluated 19 different CNNs (with a wide variety of architectures and sizes) and 2 classical machine learning approaches. We compared results directly against two different research technicians both on general classification and a more fine-grained recognition task (different brain planes). We also evaluated different components and training/test protocols, to pinpoint areas that need improving, promoting further research.

As main limitation of the study, we realize that many other methods could have been benchmarked, and that computational model’s could have benefited from the application of previous steps such as image segmentation instead of analyzing images as a whole (especially in the case of fine-grained brain plane recognition). However, maximizing performance was not the scope of the study; by making the dataset public we hope to promote this type of research.

## Conclusions

To conclude, for the first time computational models have been shown to be mature enough for its widespread application to real maternal-fetal clinical settings, especially as support in general US recognition. However, several concerns remain about its transferability and application to fine-grained categorization, without which modern clinical diagnosis would not be possible. We hope this study and our maternal-fetal US dataset will serve as a catalyzer for future research.

## Data Availability

All data associated with this study is available in the main text. Furthermore, the entire ultrasound dataset will be made publicly available upon publication from a dedicated website, https://zenodo.org/record/3904280, together with the list of papers using the dataset, to report performance improvements.

## References

[1] Hadlock, F. Estimation of fetal weight with the use of head, body and femur measurements, a prospective study. *Am J Obstet Gynecol* **151** (1985). 10.1016/0002-9378(85)90298-4 3881966

[2] Miller J, Turan S, Baschat AA (2008). Fetal growth restriction. Semin. Perinatol..

[3] Nicolaides KH, Syngelaki A, Ashoor G, Birdir C, Touzet G (2012). Noninvasive prenatal testing for fetal trisomies in a routinely screened first-trimester population. Am. journal obstetrics gynecology.

[4] Figueras F, Gratacos E (2014). Update on the diagnosis and classification of fetal growth restriction and proposal of a stage-based management protocol. Fetal Diagn. Ther..

[5] Burgos-Artizzu, X. P., Perez-Moreno, A., Coronado-Gutierrez, D., Gratacos, E. & Palacio, M. Evaluation of an improved tool for non-invasive prediction of neonatal respiratory morbidity based on fully automated fetal lung ultrasound analysis. *Sci. Reports* **9** (2019). 10.1038/s41598-019-38576-w PMC6374419 30760806

[6] Kagan K, Sonek J (2015). How to measure cervical length. Ultrasound Obstet Gynecol.

[7] Baños N (2018). Quantitative analysis of cervical texture by ultrasound in mid-pregnancy and association with spontaneous preterm birth. Ultrasound Obstet. Gynecol..

[8] Salomon LJ (2011). on behalf of the ISUOG Clinical Standards Committee. Practice guidelines for performance of the routine mid-trimester fetal ultrasound scan. Ultrasound Obstet. Gynecol..

[9] Sovio U, White IR, Dacey A, Pasupathy D, Smith GC (2015). Screening for fetal growth restriction with universal third trimester ultrasonography in nulliparous women in the pregnancy outcome prediction (pop) study: a prospective cohort study. The Lancet.

[10] LeCun Y, Bengio Y, Hinton G (2015). Deep learning. Nat..

[11] Esteva A (2017). Dermatologist-level classification of skin cancer with deep neural networks. Nat..

[12] Hosny A, Parmar C, Quackenbush J, Schwartz LH, Aerts HJ (2018). Artificial intelligence in radiology. Nat. Rev. Cancer.

[13] Litjens G (2017). A survey on deep learning in medical image analysis. Med. image analysis.

[14] Baumgartner CF (2017). Sononet: Real-time detection and localisation of fetal standard scan planes in freehand ultrasound. IEEE Transactions on Med. Imaging.

[15] Maraci M, Bridge C, Napolitano R, Papageorghiou A, Noble J (2017). A framework for analysis of linear ultrasound videos to detect fetal presentation and heartbeat. Med. Image Analysis.

[16] Ryou, H. *et al*. Automated 3d ultrasound biometry planes extraction for first trimester fetal assessment. In *Machine Learning in Medical Imaging*, 196–204 (2016).

[17] Li Y (2018). Standard plane detection in 3d fetal ultrasound using an iterative transformation network. Medical Image Computing and Computer Assisted Intervention – MICCAI.

[18] Cheng PM, Malhi HS (2017). Transfer learning with convolutional neural networks for classification of abdominal ultrasound images. J. Digit. Imaging.

[19] Robinson H (1973). Sonar measurement of fetal crown-rump length as means of assessing maturity in first trimester of pregnancy. Br. Medicine J..

[20] Appel, R., Burgos-Artizzu, X. & Perona, P. Improved multi-class cost-sensitive boosting via estimation of the minimum-risk class. *arXiv preprint arXiv:1607.03547* (2016).

[21] Dalal, N. & Triggs, B. Histograms of oriented gradients for human detection. In 2005 IEEE computer society conference on computer vision and pattern recognition (CVPR’05), vol. 1, 886–893 (IEEE, 2005).

[22] Simonyan, K. & Zisserman, A. Very deep convolutional networks for large-scale image recognition. CoRR abs/1409.1556 (2014).

[23] He, K., Zhang, X., Ren, S. & Sun, J. Deep residual learning for image recognition. CoRR abs/1512.03385 (2015).

[24] Szegedy, C. *et al*. Going deeper with convolutions. CoRR abs/1409.4842 (2014).

[25] Sandler, M., Howard, A., Zhu, M., Zhmoginov, A. & Chen, L.-C. Mobilenetv2: Inverted residuals and linear bottlenecks. *CVPR* (2018).

[26] Xie, S., Girshick, R., Dollar, P., Tu, Z. & He, K. Aggregated residual transformations for deep neural networks. In *Proceedings of the IEEE Conference on Computer Vision and Pattern Recognition*, 1492–1500 (2017).

[27] Hu, J., Shen, L. & Sun, G. Squeeze-and-excitation networks. CoRR abs/1709.01507 (2017).

[28] Huang, G., Liu, Z. & Weinberger, K. Q. Densely connected convolutional networks. CoRR abs/1608.06993 (2016).

[29] Russakovsky O (2015). ImageNet Large Scale Visual Recognition Challenge. Int. J. Comput. Vis. (IJCV).

[30] Abadi, M. *et al*. TensorFlow: Large-scale machine learning on heterogeneous systems https://www.tensorflow.org/ (2015).

[31] Vedaldi, A. & Lenc, K. Matconvnet – convolutional neural networks for matlab. In *Proceeding of the ACM Int. Conf. on Multimedia* (2015).

[32] Albanie, S. *Matconvnet pretrained models*, http://www.robots.ox.ac.uk/albanie/mcn-models.html (2018).

[33] Ioffe, S. & Szegedy, C. Batch normalization: Accelerating deep network training by reducing internal covariate shift. CoRR abs/1502.03167 (2015).

[34] Cui, Y., Jia, M., Lin, T.-Y., Song, Y. & Belongie, S. Class-balanced loss based on effective number of samples. In *Proceedings of the IEEE Conference on Computer Vision and Pattern Recognition*, 9268–9277 (2019).

[35] Winslow, R. L., Trayanova, N., Geman, D. & Miller, M. I. Computational medicine: Translating models to clinical care. *Sci. Transl. Medicine* **4** (2012). 10.1126/scitranslmed.3003528 PMC3618897 23115356

[36] Price, W. N. Big data and black-box medical algorithms. *Sci. Transl. Medicine* **10** (2018). 10.1126/scitranslmed.aao5333 PMC6345162 30541791

