# Author Correction: Evaluation of deep convolutional neural networks for automatic classification of common maternal fetal ultrasound planes

**DOI:** 10.1038/s41598-022-06173-z

**Published:** 2022-01-31

**Authors:** Xavier P. Burgos-Artizzu, David Coronado-Gutiérrez, Brenda Valenzuela-Alcaraz, Elisenda Bonet-Carne, Elisenda Eixarch, Fatima Crispi, Eduard Gratacós

**Affiliations:** 1 BCNatal - Barcelona Center for Maternal-Fetal and Neonatal Medicine (Hospital Clínic and Hospital Sant Joan de Deu, University of Barcelona), Barcelona, Spain; 2 Transmural Biotech S.L., Barcelona, Spain; 3 Institut D’Investigacions Biomediques August Pi i Sunyer, IDIBAPS, Barcelona, Spain; 4 Center for Biomedical Research on Rare Diseases (CIBER-ER), Madrid, Spain

Correction to: *Scientific Reports* 10.1038/s41598-020-67076-5, published online 23 June 2020

The original version of this Article contained an error.

The Data availability section did not include an URL link to the ultrasound dataset.

“All data associated with this study is available in the main text. Furthermore, the entire ultrasound dataset will be made publicly available upon publication from a dedicated website together with the list of papers using the dataset, to report performance improvements.”

now reads:

“All data associated with this study is available in the main text. Furthermore, the entire ultrasound dataset will be made publicly available upon publication from a dedicated website, https://zenodo.org/record/3904280, together with the list of papers using the dataset, to report performance improvements.”

The original Article has been corrected.

